# A role for the cancer-associated miR-106b~25 cluster in neuronal stem cells

**DOI:** 10.18632/aging.100302

**Published:** 2011-03-31

**Authors:** Barrie Peck, Almut Schulze

**Affiliations:** Gene Expression Analysis Laboratory, Cancer Research UK London Research Institute, London WC2A 3LY, UK

In the last decade, micro-RNAs (miRNAs) have emerged as major regulators of cell fate. They are involved in fine-tuning gene expression in normal developing tissues and are often aberrantly expressed in different disease states, including cancer. miRNAs are 20-25 nucleotide non-coding RNAs that repress the translation and stability of a large number of target mRNAs.

The study by Brett et al in the previous issue of AGING adds to our understanding of how miRNAs regulate the differentiation of adult neural stem cells (NSCs) [1]. The authors used primary cultures of neural stem/progenitor cells (NSPCs) isolated from adult mice to investigate the importance of a specific miRNA cluster, miR-106b~25, in regulating the proliferative potential and differentiation of NSCs. This miRNA cluster is located within an intronic region of the Mcm7 gene and codes for three different miRNA species, miR-106b, miR-93 and miR-25. Interestingly, activation of this miRNA cluster has been observed in different tumour types and is involved in the inhibition of anti-proliferative and pro-apoptotic genes, such as p21, Bim and TGF-beta [2,3]. Furthermore, this cluster is overexpressed in prostate cancer where it is involved in the downregulation of PTEN expression and also cooperates with its host gene Mcm7 to drive tumourigenesis [4].

The current study shows that the miR-106b~25 cluster is present in self-renewing adult NSPCs and does not change its expression when cells are stimulated to undergo differentiation. Among the three miRNAs within the cluster, miR-25 seems to be the most important for maintaining proliferation of adult NSPC. Overexpression of either miR-25, or the whole cluster, induced proliferation of NSPCs and increased the proportion of cells positive for the neuronal marker Tuj1.

Micro-RNAs are key regulators of proliferation, self-renewal and differentiation in both embryonic and adult stem cells [5]. Embryonic stem cells deplete of Dicer, an essential component of the miRNA processing machinery, fail to induce a differentiation marker upon induction of differentiation in vitro [6]. miRNAs are also involved in fine-tuning gene expression during the transition of neuronal stem cells to neuronal progenitors and neurons [7]. Some of the target genes of miRNAs involved in neurogenesis have been identified, but a large component of the complex regulatory networks, involving both positive and negative feedback loops, remains to be elucidated.

Brett et al. used a bioinformatics approach to identify potential targets of miR-25 and found over-representation of genes involved in the TGF-beta and insulin/IGF/Akt signalling pathways. The insulin/IGF/Akt signalling pathway inhibits the activity of members of the O-subfamily of forkhead-box containing transcription factors, which are important regulators of cell proliferation and survival [8]. There is clear evidence that FOXO factors are involved in neuronal stem cell maintenance. Deletion of FOXO3a alone, or combined deletion of FOXO1, FOXO3a and FOXO4, results in a decreased number of NSCs *in vivo*, and reduces their capacity for proliferation and self-renewal *in vitro* [9,10]. One study showed that loss of FOXO function causes activation of Wnt signalling and increases short-term proliferation of adult NSCs [10]. Another study observed that FOXO3a regulates the transcription of several genes associated with hypoxia response, cell cycle regulation or cell metabolism, and was able to detect FOXO3a binding to the promoters of the p27 and Ddit4 genes in adult NSCs [9].

Brett et al. found a FOXO binding site (FHRE) within the first intron of the Mcm7 gene and moreover, discovered that overexpression of a constitutively active mutant of FOXO3a resulted in increased activity of a reporter construct carrying this genomic region. However, when they investigated the expression of the three miRNAs encoded by the miR-106b~25 cluster in NSPCs from wild type or FOXO3a-null mice, they found, somewhat surprisingly, that their expression was increased rather than decreased. This result suggests a complex interrelationship between transcriptional activation of this locus and the expression of the miRNAs embedded within it.

FOXO3a has been shown to be both a regulator and a target for miRNAs in different cell types. For example, FOXO3a can repress the expression of miR-21, a suppressor of the pro-apoptotic gene Fas Ligand (FasL) in human lung cancer cells [11], but downregulation of FOXO3a by miR-155 contributes to cell survival, growth and resistance to chemotherapy in breast cancer cells [12]. One possible explanation as to why FOXO3-null NSPCs did not display a reduction in miR106~25 expression could be compensation from other FOXO family members, such as FOXO1. Also, the regulation of miR-106b~25 by FoxO3a could be dependent on recapitulating the exact physiological setting encountered by NSCs *in vivo*, such as the hypoxic conditions often associated with the stem cell niche. Then again, the regulation of miR-106b~25 by FOXO3a could be indirect. Interestingly, Mcm7 is a transcriptional target for the N-Myc oncogene in neuroblastoma [13]. Several studies have shown that FOXO factors can inhibit Myc dependent transcription through different mechanisms, including micro-RNA mediated regulation [14,15,16]. It could be interesting to investigate whether Myc family members are involved in the regulation of neurogenesis by the miR-106b~25 cluster.

There is now increasing evidence that adult stem cell maintenance is part of the increasing collection of FOXO functions related to aging. This is particularly compelling in the light of the identification of single nucleotide polymorphisms (SNPs) within the FOXO3a gene that show strong association with longevity [17]. FOXO factors balance stress resistance, cell proliferation and survival in many cell types. Disrupting the proliferation and self-renewal capacity of adult stem cells is likely to have detrimental consequences, and could contribute to complex disease states. Understanding the exact role of FOXO factors and micro-RNAs in stem cell biology will be important for the understanding of the basic process of aging, as well as age-related diseases, such as type 2 diabetes, Alzheimer's disease and cancer.
